# The Social Effects of Exergames on Older Adults: Systematic Review and Metric Analysis

**DOI:** 10.2196/10486

**Published:** 2018-06-28

**Authors:** Jinhui Li, Mojisola Erdt, Luxi Chen, Yuanyuan Cao, Shan-Qi Lee, Yin-Leng Theng

**Affiliations:** ^1^ Centre for Healthy and Sustainable Cities Nanyang Technological University Singapore Singapore; ^2^ Centre for Family and Population Research National University of Singapore Singapore Singapore

**Keywords:** active video games, psychosocial well-being, ageing, literature review, citation analysis

## Abstract

**Background:**

Recently, many studies have been conducted to investigate the effects of exergames on the social well-being of older adults.

**Objective:**

The aim of this paper is to synthesize existing studies and provide an overall picture on the social effects of exergames on older adults.

**Methods:**

A comprehensive literature search with inclusive criteria was conducted in major social science bibliographic databases. The characteristics of exergames, participants, methodology, as well as outcome measurements were extracted from the relevant studies included in the review. The bibliometric and altmetric outreach of the included studies were also investigated.

**Results:**

A total of 10 studies were included in the review, with 8 studies having used the Nintendo Wii platform. Most of the studies recruited healthy older adults from local communities or senior activity centers. Three groups of social-related outcomes have been identified, including emotion-related, behavior-related, and attitude-related outcomes. A metric analysis has shown that the emotion-related and behavior-related outcomes received high attention from both the academic community and social media platforms.

**Conclusions:**

Overall, the majority of exergame studies demonstrated promising results for enhanced social well-being, such as reduction of loneliness, increased social connection, and positive attitudes towards others. The paper also provided implications for health care researchers and exergame designers.

## Introduction

Significant population aging has been experienced by countries worldwide. In 2015, a report from the United Nations [1] indicated the number of older adults aged 60 and above was 901 million, which equated to 13% of the entire population. The report predicted that this number is expected to double by 2050, reaching nearly 2.1 billion people. Population ageing has become a major global demographic trend and has subsequently raised many public concerns on the well-being of older adults [2,3]. Older adults often suffer from several common negative events, such as a lack of close family ties (eg, living alone), loss of a loved one, a decline in mobility, or a reduction in active participation in social activities. The accumulation of these negative events could result in inadequate social support or impaired social interaction [4,5]. Some studies have indicated that a lack of social interaction led to frequently experienced social problems and disorders, such as social isolation and loneliness [6-8]. Given the potential harmful effects of social isolation and loneliness, it is important to develop social interventions to reduce emotional damage to older adults and inappropriate health and social service usage.

With the advent of digital technology, exergames, which combine digital gaming and physical exercise, are commonly used as daily exercise programs [9]. Despite being originally designed for entertainment, exergames are increasingly used for health promotion. There is a rapid growth in the popularity and use of exergames as health programs in public settings, such as in communities [10], school, and work environments [11]. Many previous studies [12,13] have assessed the potential benefits of exergames on participants’ physical, cognitive, and psychological well-being. For example, evidence from a 2-week pilot study demonstrated that exergames were able to significantly improve upper extremity function for poststroke patients [14]. A pilot study from Chan et al [15] showed that older adults in virtual reality cognitive training programs had better improvements in repetition and memory retention than those in usual programs. Albores et al [16] reported that older patients with chronic obstructive pulmonary disease showed significant improvements in their emotional well-being after a 12-week Wii Fit training program.

The social effects of exergames have drawn considerable attention from researchers [17-19]. Exergaming is a social experience which gives the players opportunities to interact with each other. This can in turn foster social networking and friendships among the players. Results from a study published by Kooiman and Sheehan [17] showed that exergaming over the internet increased students’ social relatedness in physical education. Social interaction was reported as the most important motivation for adolescents in a 20-week exergaming intervention [20]. In addition to the positive effects experienced by the younger generations, recent research on exergames has also extended to investigate of the social effects of exergaming to the older population [21,22].

Considering the major concerns regarding older adults with social disorders, it is important to have an overview on whether exergames may serve as an effective intervention for the social well-being of this group of people. In the literature on exergaming, many systematic reviews examined only on the physical and cognitive benefits of exergaming on older adults [23-25]. Some studies have reviewed the psychosocial effects of exergames, however, these studies are focused on psychological changes, such as the effect of exergaming on depression, mood, and enjoyment of exercise [26-29]. Therefore, an overall picture on the social effects of exergames on older adults is required. With increasing research efforts in the field of exergaming, the current systematic review was conducted with the aim of synthesizing the existing literature and to provide implications for improving social well-being in older adults using exergaming. Additionally, the review also investigated the bibliometric and altmetric outreach of the included studies in this systematic review, to understand their impacts in both academic and nonacademic (social media) platforms.

## Methods

The current review adopted the definition of an exergame from Oh and Yang [9] who defined it as “an experiential activity in which playing exergames or any videogames requires physical exertion or movements that are more than sedentary activities and also include strength, balance, and flexibility activities.” The studies included in the systematic review should thus involve exergames, according to the above definition, as the primary intervention of the study. Other inclusion criteria were: (1) the study should incorporate measures of social outcomes such as social connection, social bonding, or loneliness; (2) the study should target participants aged 55 or above; (3) the study should report original research in English. The term “older adult” commonly refers to a person having a chronological age of 65 years and older [30] but extensive studies indicate that insights into the needs of future older adults could be provided if pre-elderly adults aged 55 to 64 were included in the investigations [31,32]. Therefore, the current review included studies with participants aged 55 and above. The current review has a restriction to English-only articles because non-English publications do not appear in major bibliographic databases. In order to achieve a complete picture of exergaming effects on social outcomes, there were no constraining criteria applied with regard to the study design. Thus, the review included studies which used both qualitative and quantitative methods.

A comprehensive literature search was conducted in major social science bibliographic databases, including PsycINFO, PubMed, CINAHL, and ScienceDirect. Potential studies were identified by the combination of exergame terms (exergame OR Wii OR Kinect OR active video game), social terms (socia* OR social support OR social interaction OR social bonding OR communicat*), and ageing terms (aging OR aged OR elderly OR older OR senior). Reference lists of the included studies and relevant reviews were also inspected for additional studies to be included in the current systematic review. A total of 319 articles published before 22 January 2017 were retrieved for review and analysis. All articles were assessed using either the title, abstract, or full text to determine their eligibility in the systematic review conducted in this paper. The articles without full texts in any of the databases listed above excluded if the full texts could not be retrieved using online search engines or by contacting the authors directly. After identifying the final list of included studies, the characteristics of exergames, participants (country, sample size, age, and profile), methodology (study design and duration), as well as outcome measurements were extracted from the studies. Screening and data extraction was performed mainly by one reviewer, while a second reviewer was assisted by checking and editing the extracted data.

To investigate the bibliometric score (in terms of citation count), the altmetric score, and the social media presence of the articles included in the systematic review, we collected citation counts from Scopus [33], as well as usage and capture data from PlumX [34] before 31 April 2017. We also collected Tweet counts, number of Mendeley readers, and the Altmetric Attention Score from Altmetric [35] for each included article. Altmetrics can be described as new or alternative measures of the impact of research objects, based mainly on social media data sources [36]. The Altmetric Attention Score is a weighted aggregate metric comprising diverse online sources from news outlets, policy documents, blogs, Wikipedia, Twitter, Facebook, YouTube, and other social media sources. Usage data from PlumX is a combined metric incorporating counts from downloads, views, library holdings, video plays, clicks, collaborators, and other usage metrics. Capturing data from PlumX comprises counts from bookmarks, favorites, followers, readers, subscribers, watchers, exports or saves, and code forks. The 2015 QS world university rankings [37] were used to determine the prestigious universities. Prestigious universities were defined as those listed in the 2015 QS world university rankings. We used a logarithmic scale for a better visualization of the data. These metrics gave us an insight into the outreach and impact of the included studies in this systematic review.

## Results

### Study Selection

**Figure 1:**
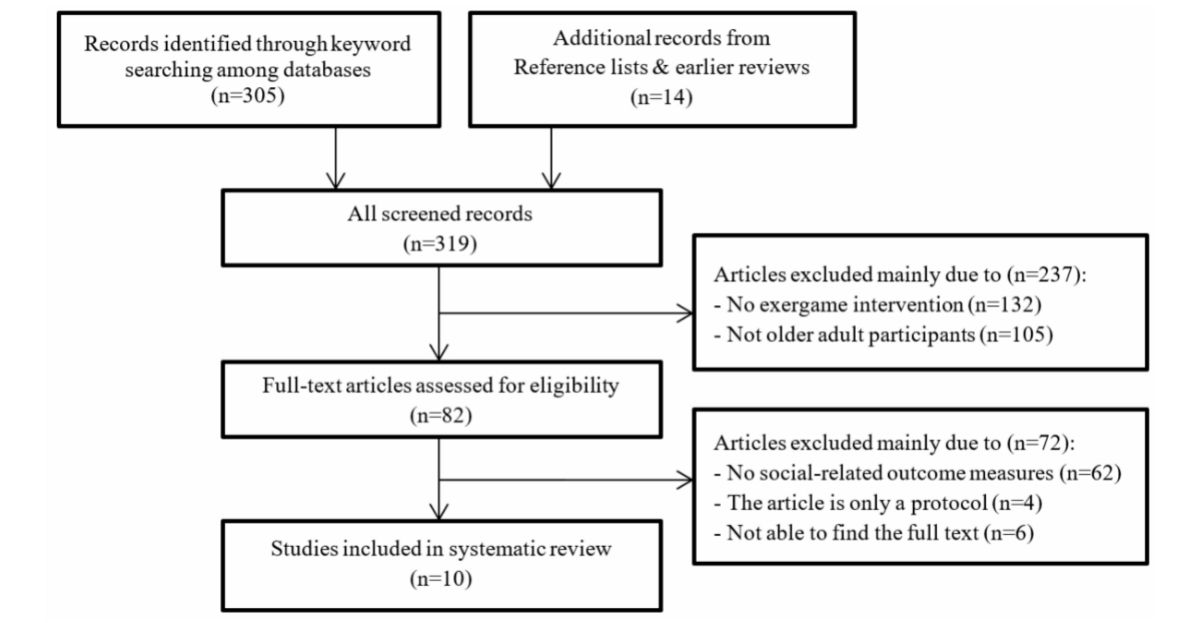
Flowchart of the systematic review process for the included studies.

According to the inclusive criteria, a total of 10 studies were eligible to be included in the final review process. Figure 1 illustrates the flowchart of the systematic review process for the selection of the included studies. Tables 1 and 2 outline the key characteristics of these 10 studies.

### Characteristics of the Studies Included

#### Exergame Types

Of the 10 studies investigated, 8 investigated social effects of exergames using Nintendo Wii, while 2 studies used Microsoft Xbox Kinect. Both platforms are the most popular exergaming platforms in the current market, and both offer console-based devices and games which make exergaming possible in the home setting. In terms of game topics, it is interesting to note that half of the studies (n=5) applied games from Nintendo Wii Sports package [39-43]. The Wii Sports game package allows participants to play virtual sport games (such as tennis, bowling, baseball, golf, or boxing) by performing body motions that they would do in actual sports. Another study from Wu, Li, and Theng [45] also used a virtual bowling game, but from the Microsoft Kinect Sports game package. Two studies used exercise games from Nintendo Wii Fit, or its successor Nintendo Wii Fit U [21,38]. Wii Fit exergames are different to Wii Sports games as they aim to improve players’ physical fitness through exercise activities such as strength training, aerobics, yoga, and balance games. Besides those simulating actual exercise, exergames with topics from daily life activities were also found in two studies [39,43], such as cookery simulation-styled *Cooking Mama* and party simulation-styled *Wii Party*.

#### Participants

Most of the studies recruited healthy older adults from local communities or senior activity centers. There were 2 studies [38,44], however, that investigated the social effects of exergames on older adults with physical or social problems, such as those with impaired balance, with a disability, or those who were socially isolated. Six studies included participants with Western cultural backgrounds, including the US, Australia, and Canada. Among the studies focused on Western cultural backgrounds, one study focused on African American participants [21]. Four studies were conducted in the context of Asia, all of which were conducted in Singapore. The majority of the studies had a small sample size with less than 50 participants.

#### Methodology

Five of the 10 studies applied poststudy qualitative methods to assess the social effects of exergames, such as semistructured interview, semistructured group interview, or focus group discussion. Among those applying quantitative methods, 4 studies tested the effects between exergames and other control conditions. For example, Wu et al [45], Jung et al [39], and Kahlbaugh et al [40] compared exergames with traditional activities such as playing board games, watching television programs, or performing normal exercise. Xu et al’s [22] study is an exception which compared the effects among 3 exergame conditions (playing alone vs playing with elderly vs playing with youths). One study [43] applied a within-group experiment method to compare the effects before and after the exergame intervention. The duration of the intervention period ranged from 1 to 12 weeks, while most of the studies involved 8 sessions or more. Two reviewers independently applied the risk of bias tool from Cochrane Collaboration [46] to assess the methodological quality of 4 studies with control conditions. Table 3 shows the results of the quality assessment of the 4 controlled studies. According to the Cochrane recommendations [46], 2 studies [22,45] were identified to have “High Risk of Bias,” while the other 2 studies [39,40] were identified to have “Moderate or Unclear Risk of Bias.”

### Social Outcomes

The findings of the included studies have identified several social-related outcomes. Based on the different natures of the outcomes, they were categorized into three groups: emotion-related, behavior-related, and attitude-related.

#### Emotion-Related

Loneliness was identified to be the main emotion-related social outcome affected by exergames. Jung et al conducted a study to assess the potential of Nintendo Wii in improving the quality of life among older adults in a long-term care facility [39]. Their results indicated that elderly participating in the Wii condition group had a significantly lower level of loneliness than those participating in the other condition group, who played traditional board games. Similarly, another between-group study in the US also reported that playing Wii rather than watching television programs led to a lower level of loneliness [40].

Additionally, Xu et al found a significant decrease in loneliness among older adults after exergaming, although little differences were found across different play types or age groups (young-old vs old-old) [22]. In the same study, social anxiousness was also found to have significantly declined, however, this was noticed only in the young-old participants who played exergames with youths [22].

**Table 1:** Characteristics of the included studies (exergames and participants).

Study	Exergames	Participants			
		Country	Sample size	Age in years	Profile
Agmon et al [38]	Wii Fit Exergames (basic step, soccer heading, ski slalom, and table tilt)	US	7	84 (5)^a^	Older adults with impaired balance From care retirement communities
Chao et al [21]	Wii Fit U (balance games, yoga poses, strength training, aerobics, and dance games)	US	12	64.17 (6.74)^a^	Healthy older female adults aged 55 years and above From African American community
Jung et al [39]	Wii Sports (tennis, bowling, baseball and boxing) and Cooking Mama	Singapore	45	56-92	Local healthy older adults From senior activities centers
Kahlbaugh et al [40]	Wii game (Wii bowling)	US	35	82 (9.8)^a^	Healthy older adults From independent living residential apartments
Keogh et al [41]	Nintendo Wii Sports	Australia	34	83 (8)^a^	Healthy older adults From residential aged care centers
Millington [42]	Exergame such as Wii Bowling	Canada	8	N/A^b^	Healthy older persons From retirement centers
Theng et al [43]	Wii games such as “Wii Sports,” “Cooking Mama,” and “Wii Party”	Singapore	28	>60	Healthy older adults From a seniors’ activity center
Wollersheim et al [44]	Wii games	Australia	11	73.5 (9)^a^	Older women with a disability or who are socially isolated
Wu et al [45]	Kinect Sport Bowling with a partner	Singapore	113	>55	Healthy old adults From senior activity centers and community clubs
Xu et al [22]	Three Kinect exergames	Singapore	89	75	Local healthy older adults From senior activities centers

^a^Age presented as mean (SD).

^b^N/A: not available.

**Table 2:** Characteristics of the included studies (methodology and outcome).

Study	Methodology		Outcome	
	Study design^a^	Duration	Measurement	Effect
Agmon et al [38]	Within-group, poststudy interview	3 sessions per week; 12 weeks	Socialization: semistructured interview	Six out of 7 participants described that they enjoyed playing Wii Fit with their grandchildren
Chao et al [21]	Within-group, poststudy interview	2 sessions per week; 12 weeks	Social connection: semistructured interviews	The program encouraged participants to get connected with others
Jung et al [39]	Between-group, 2 conditions: Playing exergames (N=30) Playing traditional board games (N=15)	3 sessions per week; 6 weeks	Loneliness: UCLA^b^ Loneliness Scale	Exergame group versus control group: *t*_43_=5.34, *P*<.01
Kahlbaugh et al [40]	Between-group, 3 conditions: Playing exergames with a partner (N=16) Watching television programs with a partner (N=12) No visits (N=7)	1 session per week; 10 weeks	Loneliness: UCLA Loneliness Scale	Exergame group versus television group: *F*_2,30_=6.24, *P*<.005
Keogh et al [41]	Within-group, poststudy interview	8 weeks	Socialization: semistructured group interview	“Several (P5 and P4) found that having a ‘new face’ to interact with and someone who would sit and listen was something to look forward to.”
Millington [42]	Within-group, poststudy interview	Wii constant use at one center; 1 to 2 times per month for another two centers	Social engagement: Interview	“Virtual bowling can bring people together in communal spaces while also ‘getting them up’ and active”
Theng et al [43]	Within-group, pre- and poststudy measurement	6 sessions	Positive attitude: semantic differential scale	Mean positive attitude towards youth: increased from 4.06 (SD 0.78) to 4.27 (SD 0.43)
Wollersheim et al [44]	Poststudy focus group discussion	2 sessions per week; 6 weeks	Social bonding: focus group discussion	“Many of the women noted that being more technologically adept allowed them to be more connected to their grandchildren.”
Wu et al [45]	Between-group, 4 conditions: Playing collaborative exergame (N=26) Playing competitive exergame (N=24) Playing collaborative traditional exercise (N=25) Playing competitive traditional exercise (N=20)	2 sessions per week; 4 weeks	Social presence: The Social Presence in Gaming questionnaire	Exergame group versus traditional exercise group: beta=–.20, *P*<.10 (in general intention model)
Xu et al [22]	Between-group, 3 conditions: Playing exergames with their peers (N=31) Playing with an adolescent (N=26) Playing alone (N=31)	3 sessions per week; 1 week	3 measurements: Loneliness: UCLA Loneliness Scale Social anxiousness: the interaction anxiousness scale Sociability: sociability scale	3 effects: Loneliness: significantly decreased after playing exergames, *F*_1,83_=.57, *P*<.05 Social anxiousness: did not change significantly, *F*_1,83_=1.58, *P*=.212 Sociability: significantly increased after playing exergames, *F*_1,83_=3.95, *P*=.050

^a^For social outcomes.

^b^UCLA: University of California, Los Angeles.

**Table 3:** Results of quality assessment of four controlled studies included in the review.

Citation	Random sequence generation	Allocation concealment	Blinding of participants and personnel	Blinding of outcome assessment	Incomplete outcome data	Selective reporting	Other bias
Jung et al [39]	Low bias	Unclear	Unclear	Unclear	Low bias	Low bias	Low bias
Kahlbaugh et al [40]	Low bias	Unclear	Unclear	Unclear	Low bias	Low bias	Low bias
Wu et al [45]	High bias	High bias	Unclear	Unclear	Low bias	Low bias	Low bias
Xu et al [22]	High bias	Unclear	Unclear	Unclear	Low bias	Low bias	Low bias

#### Attitude-Related

Wu et al [45] presented a study which examined the exergame effects on social presence, which was defined as the sense of connecting or being with others in a media-mediated environment. Their results found that older adults in the exergame setting had a significant lower social presence than those in traditional exercise. Another Singapore study from Theng et al [43] showed that playing exergames with youths led to improvement in older adults’ positive attitude toward the younger age group.

### Metric Analysis

**Figure 2:**
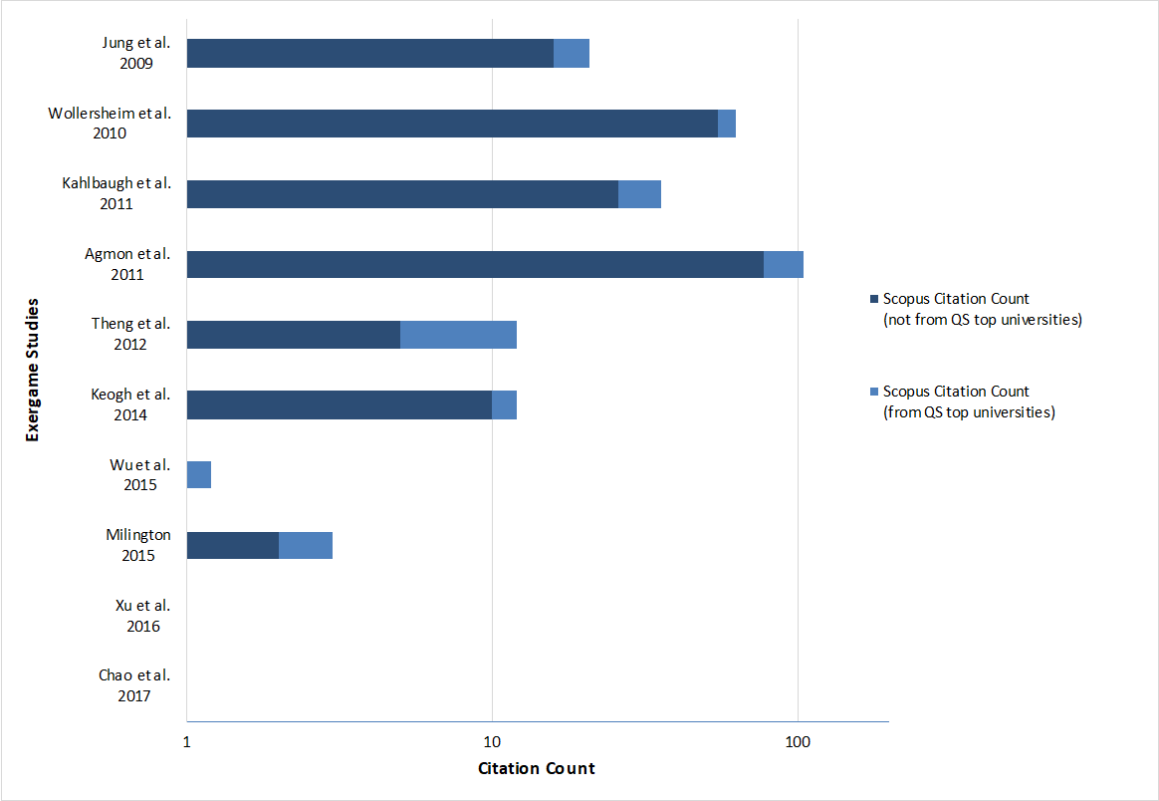
Overview of the bibliometric outreach of included studies.

Currently, the study from Agmon et al [38] has received the most attention from the scholarly community to date, with a total of 105 citations, 27 of which came from prestigious universities. The study published by Wollersheim et al [44] has also received a good amount of attention with a total of 63 citations, of which 8 were from prestigious universities. Most of the citations of these two studies came from articles published between 2014 and 2016, and mainly from papers published in the fields of Medicine and Computer Science. Since the studies from Xu et al [22] and Chao et al [21] were only recently published (they were published in December 2016 and January 2017 respectively), no citations could yet be found for these articles. Figure 2 shows the bibliometric outreach of the exergame studies.

**Figure 3:**
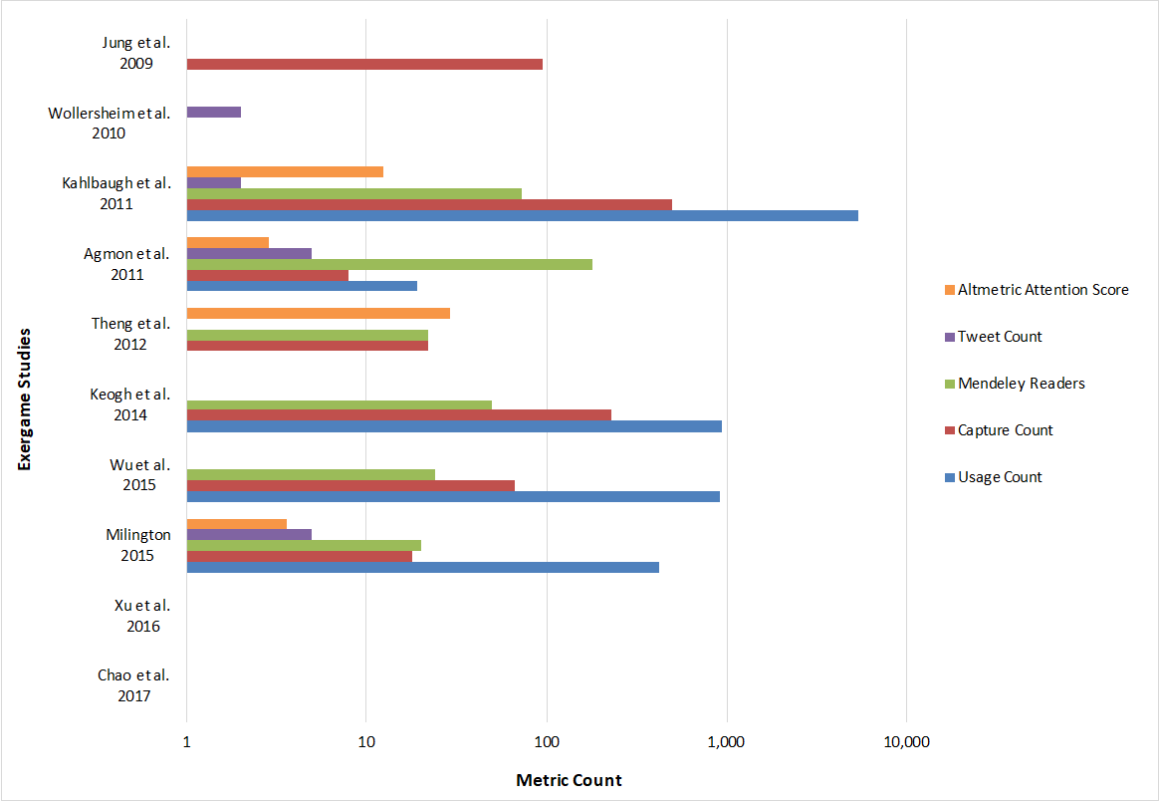
Overview of the altmetric outreach of included studies.

Figure 3 gives an overview of the altmetric outreach of the exergame studies. The study with the highest Altmetric Attention Score was Theng et al with a score of 29 [43]. This high score was attributed to 3 mentions on news outlets in March 2017, naming this study as an example of how Nintendo’s motion control system has helped to make gaming accessible to new groups of users. Kahlbaugh et al had a very high PlumX usage count of 5300, and a high PlumX capture count of 498 [40]. These were mainly due to abstract views, clicks on outbound links, and exports or saves on EBSCO [47].

Agmon et al had a high count of 180 Mendeley readers, but since Altmetric.com does not include Mendeley readers in its score, this is not reflected as this study had a score of 3 [38]. Again, two recent studies [21,22] did not yet have any altmetrics, however, one tweet could already be found for the article from Chao et al [21], giving this article an Altmetric Attention Score of 0.5. Tweet counts were however low across all studies, with Milington [42] and Agmon et al [38] having the highest count of 5 tweets each.

## Discussion

### Principal Findings

While previous reviews have synthesized the psychosocial effects of exergames [26,28,29], the current review focused specifically on social benefits and extended to the ageing population. The systematic review shows an increasing interest in using exergames to improve the social well-being among older adults, with 9 out of 10 included studies published after the year 2010. Nevertheless, the small number of identified studies also calls for more investigation in this new research area. Several systematic reviews had similar findings in the area of exergames for mental health, with 12 and 9 studies found in the reviews published by Spek et al [48] and Li et al [28] respectively. Overall, the majority of exergame studies demonstrated promising results for enhanced social well-being in older adults, such as a reduction of loneliness, an increase in social connection, and positive attitudes towards others.

### Social Benefits of Exergames

Although the social benefits of exergames are often discussed in the literature on exergames, there has been no particular review found on this topic. By summarizing the existing original studies, the findings from the current review showed that exergames could be an effective intervention for social improvements among older adults. The review supported the finding that exergames were able to reduce the loneliness level among older adults. The decrease in loneliness was perhaps not due to playing the exergame itself, but rather due to the increased interactions between the participants and other players. In a large sample survey study, Lee and Ishii-Kuntz [49] indicated that doing an activity with other people reduced loneliness among older adults. Furthermore, many of the included studies suggested that exergames provide opportunities for social interaction and connectedness with peers and family members [38,41,44]. In addition to loneliness, older adults often lack the motivation to engage in exercise. Chao et al indicated that these behavior-related social outcomes of exergames may increase exercise motivation and adherence among older adults [27]. A metric analysis has shown that the emotion-related and behavior-related outcomes of exergames received a lot of attention in the academic community as well as on social media.

Attitude-related social outcomes are a new finding which have not been reported in previously published exergame reviews (eg, those published by Chao et al [27] or Matallaoui et al [29]). Exergames were found to affect a sense of being with others [45] and positive attitudes towards others [43], similar to findings in recent research on persuasive video gaming [50,51]. An experiment from a study published by Alhabash and Wise [50] found that video game role-play led to a change in students’ explicit and implicit attitudes toward Palestinians and Israelis. In another study, students who played the persuasive social impact game had an increased positive attitude towards the homeless [51]. Results from this review further supports that active video games, such as exergames, have the potential to affect older adults’ attitudes towards other groups of people. Although attitude-related social outcomes currently have a low academic impact in the exergaming research area, they have begun to receive a certain amount of discussion on social media.

### Implications for Future Study

This review showed that that Nintendo Wii was the most frequently used exergaming platform in the included studies. This finding is supported by another review [27], which reported that the Nintendo Wii is one of the most accessible and popular exergames for seniors. Chao et al [27] further indicated the high attendance rates among older players in Wii exergames programs. Although the evidence may suggest Wii to be a suitable platform for older adults to perform exergames, there have been no studies conducted which investigate the difference in effect between exergaming using a Wii and other platforms, such as the Microsoft Kinect consoles. More studies are needed to compare the effects of different exergaming platforms. Sport games were identified to be the favored type of games used in the included studies, and bowling was tested in 3 studies [40,42,45]. According to the American College of Sports Medicine [52], older adults are encouraged to perform physical activities that maintain or increase their balance and flexibility through slow movements. The bowling games exergames allow for slow movements which match the typical physical activities recommended for older adults. Additionally, bowling is a self-paced exercise in which older adults could take the time they needed to perform the moves [27]. Crucially, all the studies applied commercial exergames available on the market and none of the interventions were integrated with social theories. This highlights the need to combine social-related theories with exergame programs in order to optimize the effectiveness of social improvements.

Although most of studies targeted healthy older adults, two studies examined the social effects on older adults with physical disabilities [38,44]. Physical disability, particularly low mobility, has often been identified as a risk factor for social isolation among older adults [53]. Low mobility prevents seniors from participating in active social engagement and connection, leading to common social disorders such as loneliness. The two included studies with older adult participants with physical disabilities showed that playing exergames improved their social well-being by increasing social bonding with their peers and grandchildren. However, the physical limitations of this group of older adults may have led to some difficulties in interacting with the exergames. They may have been exposed to frustrating experiences or even accidents if the exergames were performed without proper human or technical assistance. As a result, health care providers and exergame designers should take this into consideration when implementing future social exergame programs for older adults with disabilities. In terms of cultural background, the studies were conducted in both Western and Asian contexts. It appears that exergames might have social effects on older adults with various cultural backgrounds, but knowledge is lacking on whether the social outcomes would be affected by cultural factors. Future studies are recommended to compare the social effects between different cultural contexts.

The study designs included in this study varied in rigor, with 6 studies applying a within-group design, and 4 studies applying between-group design by comparing exergames with a control condition. Although the majority of studies showed promising results for the use of exergames for social enhancement, the conclusions need to be interpreted with caution due to the limited number of randomized controlled trials. The included studies were either predominantly small pilot trials or feasibility studies; they lacked the adequate sample sizes needed for a powered efficacy trial. Meanwhile, half of the studies used qualitative methods for data collection. Without validated quantitative instruments, their findings do not have the capacity to detect significant changes in social outcomes.

### Limitations

There are some limitations in the review. Due to the limited number of identified studies, the systematic review included articles with both qualitative and quantitative analyses. The quality assessment of the included studies was difficult to conduct, and it was not possible to produce mean effect sizes via a meta-analysis. However, the review was broad in scope and included a diversity of study conditions and social outcome measures. Another limitation is that the key conclusions should be interpreted with caution due to the small number of included studies. Furthermore, relevant studies may have been unintentionally excluded because of the specific keywords used and the databases selected. Lastly, a publication bias, particularly language bias, might have occurred because we restricted the search to English language publications.

## Abbreviations

UCLA: University of California, Los Angeles
